# Self-Organization and Regulation of Intrinsically Disordered Proteins with Folded N-Termini

**DOI:** 10.1371/journal.pbio.1000591

**Published:** 2011-02-15

**Authors:** Philip C. Simister, Fred Schaper, Nicola O'Reilly, Simon McGowan, Stephan M. Feller

**Affiliations:** 1 Cell Signalling Group, Weatherall Institute of Molecular Medicine, John Radcliffe Hospital, University of Oxford, Oxford, United Kingdom; 2 Department of Systems Biology, Otto-von-Guericke-University Magdeburg, Magdeburg, Germany; 3 Peptide Synthesis Laboratory, Cancer Research UK London Research Institute, London, United Kingdom; 4 Computational Biology Research Group, Weatherall Institute of Molecular Medicine, John Radcliffe Hospital, University of Oxford, Oxford, United Kingdom; Brandeis University, United States of America

## Abstract

How do mostly disordered proteins coordinate the specific assembly of very large signal transduction protein complexes? A newly emerging hypothesis may provide some clues towards a molecular mechanism.

## Summary

Here we hypothesize that some proteins use their structured N-terminal domains (SNTDs) to organize the remaining protein chain by means of intramolecular interactions, so generating partially condensed proteins. This model has several attractive features: as the nascent protein chain emerges from the ribosome, the SNTD folds spontaneously and then serves as a nucleation point for the yet unstructured amino acid chain, creating more compact shapes. This reduces the risk of protein degradation or aggregation. Moreover, an interspersed pattern of SNTD-docked regions and free loops can coordinate assembly of sub-complexes in defined loop-sections and enables novel regulatory mechanisms, for example through posttranslational modifications of docked regions.

## Introduction

Proteins are generally thought to be made up of one or several domains composed of α-helices and/or β-strands that form spontaneously or fold with the help of chaperones. However, many proteins lack recognizable domains along much of their chains. Such proteins have been called unstructured or “intrinsically disordered” (ID) [1]. Are all of these proteins really without any structure, or is structure something that can in certain cases form by some yet unrecognized process of nucleation? Approximately one-third of human proteins appear to fall into the ID protein category [2] (see also the database of disordered proteins, DisProt [3]). Most are excluded from detailed ultrastructural analyses, as they are often considered to be poor subjects for X-ray crystallography or other structural biology techniques, so relatively little is known about their shapes and conformations or conformational changes that presumably occur during their interactions with other proteins. Nevertheless, ID proteins have important functions in multi-protein complex assembly and cell signalling [4]–[7], and we need to learn much more about their molecular activities and mechanisms of action as well as their structures.

The abundance of ID proteins in cells is somewhat puzzling, raising questions regarding their escape from proteolytic degradation and the lack of aggregate formation—the common fates of poorly folded proteins. Misfolded proteins that escape destruction are well known to cause several major neurodegenerative disorders and other “amyloid” protein deposit diseases [8]–[12]. In fact, almost all proteins contain segments that can, in principle, form amyloids [13]. Therefore, poorly folded proteins are typically targets for fragmentation by the proteasome and other proteases [14]–[16]. Structural disorder appears to serve only as a weak signal for intracellular protein degradation, however. Neither do ID proteins display an overall preference for chaperone binding in vivo [17], despite the prominent role that chaperones play in supporting protein folding in general [18]. At least some, if not many, ID proteins may therefore adopt types of order that are not easily recognized by current secondary or tertiary structure prediction programmes, which primarily recognize α-helices and β-strands and higher order assemblies built from these. Examples of secondary structure elements that are usually not detected include the poly-proline type II (PPII) [19],[20] and 3_10_ helices [21],[22]. Despite their abundance in human proteins, new examples of these helices often become apparent only through focussed structural analyses of individual proteins.

Beyond the occurrence of these often short, helical regions not detected by current structure prediction programs, it appears likely that several other molecular mechanisms generate order within unfolded protein chains, some of which may still remain to be studied in any detail. Some interesting mechanistic routes that allow the generation of defined structural states from a disordered conformation have recently been described. For example, some ID proteins adopt specific conformations in parts of their amino acid chain upon binding their partner proteins. This can go as far as adopting multiple distinct conformations depending on which of several binding partners is involved [23]. Another example are the recently proposed “disordered domains”, which are stretches longer than 20–30 amino acid residues that are thought to present functional units for protein interactions [24]. Here, we propose another mechanism by which long ID proteins might rapidly establish a degree of order within their polypeptide chains. This “N-terminal folding nucleation” (NFN) hypothesis provides a testable conceptual framework that could explain how some of the so-called ID proteins might fold to fulfil their functions in cells.

## The N-Terminal Folding Nucleation Hypothesis

**Figure 1:**
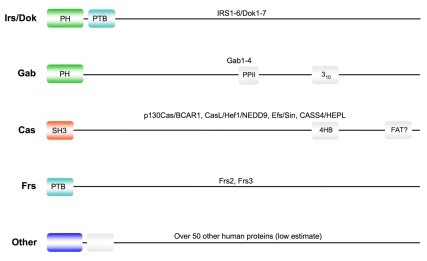
Schematic structures of selected large multi-site docking (LMD) protein families involved in signalling. The Irs/Dok, Gab, p130Cas, and Frs families of LMD proteins provide platforms for assembly of elaborate multi-protein complexes (also known as “signalosomes”) associated with a wide range of cell membrane receptors involved in regulating cell survival, growth, motility, and/or differentiation. They all have a structured N-terminal domain (SNTD) followed by an apparently largely unstructured polypeptide chain. In some cases, short secondary structure motifs like PPII helices, 3_10_ helices, etc. have been found or are suspected. Many human proteins are predicted to have a similar structural composition (for further details see Figure S2).

Large multisite docking (LMD) proteins like the Gab, p130Cas, and IRS family proteins [5],[6] facilitate the assembly of enormous signal transduction protein complexes. Such multi-protein complexes are thought to integrate and process multiple inputs from various upstream signal transducers to regulate cell survival, proliferation, cytoskeletal structures, migration, and/or differentiation. Several families of LMD proteins have a strikingly similar structural composition: a structured N-terminal domain (SNTD), for example an SH3, PH, or PTB domain, followed by a long and, according to secondary structure prediction programs, largely disordered protein chain (Figure 1). Initially, this appeared to be simply a curious feature without any obvious functional explanation, until another unexpected and seemingly unrelated finding suggested a possible rationale for this peculiar LMD protein composition. In a human embryonic kidney cell line that has intrinsically high phosphatidylinositol 3-kinase (PI3K) activity, the full-length Gab1 protein was found in the cytoplasm rather than at the plasma membrane [25]. This was surprising because the N-terminal PH domain of Gab1 binds PIP3 [26], the membrane-embedded product of PI3K, so one might have expected Gab1 to be localized at the plasma membrane. Further studies found that in these cells an additional signal is needed to bring Gab1 to the plasma membrane where it binds PIP3 with its PH domain: a serine residue (Ser552) located far away from the N-terminal PH domain in the disordered tail of Gab1 must become phosphorylated, for example by the Mek–Erk kinase module [25]. This suggests that a distant part of Gab1 binds, either directly or indirectly, to its N-terminal PH domain, thereby blocking the PIP3-binding pocket. Furthermore, this interaction occurs in a functionally regulated, cytokine signal-dependent manner.

To analyze further the interaction between the Gab1 PH domain and Ser552, we employed a peptide array overlay assay [27]–[29], in which a series of overlapping peptides corresponding to the entire Gab1 protein were probed with an affinity-purified Gab1 PH domain to identify short linear Gab1 regions that may bind directly to the PH domain (Figure S1). We found several overlapping peptides, all including Ser552, that bound directly to the Gab1 PH domain probe. In addition, other peptides corresponding to several distinct regions in the Gab1 protein also bound to the PH domain probe. If some of these potential binding regions interact with the Gab1 PH domain in vivo, this domain could be thought of as a nucleation core for intramolecular binding, and hence compaction of the supposedly disordered Gab1 tail region would occur.

**Figure 2:**
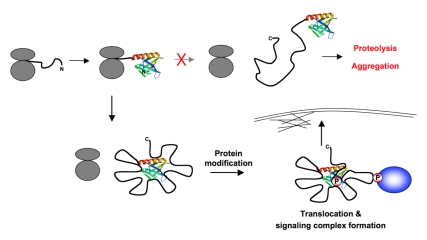
Illustration of the N-terminal folding nucleation (NFN) hypothesis. The NFN hypothesis proposes that, as the nascent chain of an LMD protein (black string) emerges from the ribosome (in grey), the SNTD folds rapidly and spontaneously and then serves as a nucleation core for additional and specific intramolecular protein chain contacts, which generate a more compact protein shape. This compaction may help to avoid proteolysis or aggregation. Instead, the arrangement of docked regions and loops generates defined regions in the protein that may serve as functional subunits. Protein modifications like phosphorylation in some of these defined regions may lead, for example, to the liberation of docked regions, allowing the SNTD to engage in novel types of interactions that might allow the anchorage of the LMD protein in specific subcellular locations. Other modifications are well known to generate docking points for interaction domains of signalling partner proteins, presumably resulting in the rapid assembly of defined sub-complexes on specific loops. Taken together, these features might be expected to increase the ability of cells to respond rapidly and selectively to a diverse set of incoming stimuli.

In addition to providing a mechanistic concept for Gab1 compaction, this NFN hypothesis also suggests a simple explanation for how some other disordered proteins might escape protein aggregation or degradation, by using a co-translational folding mechanism that differs substantially from the classical folding mechanisms used by structured proteins. As the first N-terminal amino acid residues of the polypeptide chain emerge from the ribosome, the secondary structural elements form spontaneously and rapidly fold into a highly stable SNTD. Once this is assembled, further residues emerging from the ribosome dock onto specific SNTD patches, thereby preventing the unstructured chain from engaging in nonspecific interactions and also preventing those patches on the SNTD from binding to other polypeptides in the cell (Figure 2). Moreover, the intramolecular attachment of segments of the nascent polypeptide to the SNTD would generate defined loops that may serve as docking regions for the assembly of specific sub-complexes with protein compositions that are distinct from those attached to other loop regions. In the case of Gab1, one loop may, for example, function primarily as a docking region for Crk family adaptors, while another loop may be dedicated to interacting with SHP2 phosphatase molecules.

Consistent with this idea, clustering of specific Src homology 2 (SH2) domain protein-binding sites in LMD proteins of the Gab family was noted years ago [30]. This clustering is presumed to contribute to the spatial organization of a complex's components, i.e., the quaternary structure. Six putative binding sites for the SH2 domain of the CRKL protein are located in a central region of approximately 150 amino acid residues of Gab1 (aa 259–409 in human Gab1), whereas the remaining residues lack a single putative binding motif. This clustering of CRKL-binding sites in Gab1, combined with the ability of the CRKL adaptor protein to dimerize and possibly tetramerize [31], suggests that LMD proteins like Gab1 could enable the assembly of highly ordered and stable complexes.

Clearly, these concepts are speculative and need experimental validation. They will, however, guide the design of new experiments to define the mechanisms whereby distinct and very large signal transduction complexes (also known as stimulus-specific “signalosomes”) assemble rapidly in response to diverse stimuli. The coordinated assembly of well-ordered signalling sub-complexes that can be differentially combined depending on the biological context is appealing. It would allow the speedy generation of specific signals in discrete regions of an LMD protein, which must be very desirable for at least some signalling systems. In the case of Gab1–CRKL complexes, which are prominently linked to cell shape change and motility signals through the activation of Rho family GTPases, it is easy to imagine multiple biological contexts where the ability to move swiftly would be advantageous.

Another advantage of discrete regions docking onto the SNTD would be the generation of novel target sites for signal regulation, which may, for example, contribute to the robustness of cell signalling networks [32]. This is nicely exemplified by the Gab1 phosphorylation on Ser552. Only when there are two coincident signals—one through PI3K activation and the other one by firing of Mek–Erk kinases —will Gab1 translocate to the membrane, where further phosphorylation leads to the assembly of a complex that regulates essential cell behaviors like proliferation and cell migration.

## Towards a Solution

To estimate how common NFN might be among proteins, we initially sought to define how many proteins in the human proteome have an SNTD in combination with a long disordered tail. For this, we predicted the disordered regions and structural domains for all human proteins in the UniProt SwissProt database (http://www.ebi.ac.uk/uniprot/) using DisEMBL (http://dis.embl.de/) and SMART (http://smart.embl-heidelberg.de/), respectively. This showed that, in addition to the protein families depicted in Figure 1, over 50 further proteins display a similar structural organization (for proteins and details of bioinformatics analysis see Figure S2). Of the more than 50 proteins detected, most are known to be or pre sumed to be involved in signalling processes. These NFN candidate pro teins must now be subjected to further biochemical, biophysical, and biological analyses. Gel filtration chromatography, analytical ultracentrifugation, mass spectrometry of intact proteins, and small angle X-ray scattering (SAXS) should give some information about their molecular weights, hydrodynamic radii, and shapes. Nuclear magnetic resonance (NMR) analyses of isolated SNTDs and full-length proteins should identify residues in the SNTDs that contribute to intramolecular contacts with the ID chain. In some cases, even in vivo NMR, similar to a study conducted with bacterial FlgM, may be possible [33].

Mutations of SNTD residues implicated from NMR experiments, and of key residues in the ID tails identified by peptide array overlay blots, could then be analyzed for functional defects or effects on protein turnover or aggregation in cells. In vivo studies with knock-in mutants can subsequently investigate the systemic consequences. It will also be interesting to determine whether some of the proteins utilising NFN are additionally stabilized in their compact shapes by complex formations with other proteins, which should co-purify in stoichiometric amounts. Last not least, more computational studies on the molecular radii and properties of ID proteins, similar to those previously published for a few other examples [34],[35], are warranted.

Clearly nature has found multiple ingenious ways of compacting emerging protein chains into functional units with great efficacy. As we learn more about these mechanisms, we will also begin to understand better the fundamental principles that govern the assembly and actions of complex signalling networks and their multi-protein hubs.

## Supporting Information

Figure S1 **Gab1 peptide array overlay assay identifies potential binding sites for the PH domain.** For this assay, the full amino acid sequence of Gab1 from *Mus musculus* used in the study of Eulenfeld and Schaper [25] was chemically synthesized as an array of spots of overlapping peptides (Multipep synthesiser [Intavis], with a peptide length of 23 amino acids, sliding two residues further with each consecutive peptide), blocked with 5% nonfat dry milk in TrisHCl buffer (pH 7.5) with 100 mM NaCl and 0.1% Tween 20 added and probed initially with 4 µg/ml GST, followed by incubation with anti-GST, HRP-coupled secondary antibody, and ECL detection. No GST binding was detectable to any of the peptides (top panel). The same membrane was then re-probed with 1 µg/ml of affinity-purified GST-PH domain (bottom panel). Series of dark spots correspond to clusters of nonidentical, overlapping peptides that bind to the GST-PH probe. The red box indicates the Ser552 epitope previously implicated in regulating Gab1 PH domain binding by the work of Eulenfeld and Schaper [25]. Similar results were also obtained when DTT was included in the assay to eliminate potential artefacts from non-specific interactions of Cys residues (unpublished data).
(1.68 MB TIF)

Figure S2 **Human proteins identified as NFN candidates by bioinformatics analysis.** Schematic representation of proteins identified by the prediction of disordered regions and structural domains for all human proteins in the UniProt SwissProt database (http://www.ebi.ac.uk/uniprot/) using DisEMBL (http://dis.embl.de/) and SMART (http://smart.embl-heidelberg.de/), respectively. The two sets of predictions were compared using a custom perl script to identify proteins with a predicted domain or domains in the N-terminus (defined as the first 25% of the protein), no predicted domains in the C-terminus (defined here as the remaining 75% of the protein), and predominantly disordered (>80%) in this C-terminus. Initial hits were listed with their corresponding SMART and SwissProt data and then individually inspected to exclude, for example, transmembrane proteins. Proteins shown here clearly represent an underestimate of actual candidates in the human proteome, since, for example, proteins with additional domains in the amino acid chain following the folded N-terminus were excluded, even if several hundred disordered amino acids follow the N-terminal domain. If multiple splice variants occur, only a single representative is shown for each protein. Proteins are alphabetically listed according to the gene names following the HGNC nomenclature (July 2010; http://www.genenames.org/), identifiers below the names and SNTD designations are according to the SMART database. Protein domains and chain lengths are not drawn to scale. Values on the right side indicate the number of amino acids in each protein. Many of the NFN candidates depicted here are known or suspected to act in cell signalling. Please note that LMD proteins already depicted in Figure 1 and again found in the bioinformatic analysis (FRS2, FRS3, GAB1, GAB2, GAB3, IRS1, IRS2) are not shown again in this supporting figure.
(1.98 MB TIF)

## Abbreviations

ID: intrinsically disordered
LMD: large multisite docking
NFN: N-terminal folding nucleation
NMR: nuclear magnetic resonance
PI3K: phosphatidylinositol 3-kinase
PPII: poly-proline type II
SH2: Src homology 2
SAXS: small angle X-ray scattering
SNTD: structured N-terminal domain
