# Supplementary figures and images for: Self-Organization and Regulation of Intrinsically Disordered Proteins with Folded N-Termini
Source: PLoS Biol. 2011 Feb 15;9(2):e1000591. doi: 10.1371/journal.pbio.1000591 (PMC3039663; doi:10.1371/journal.pbio.1000591)

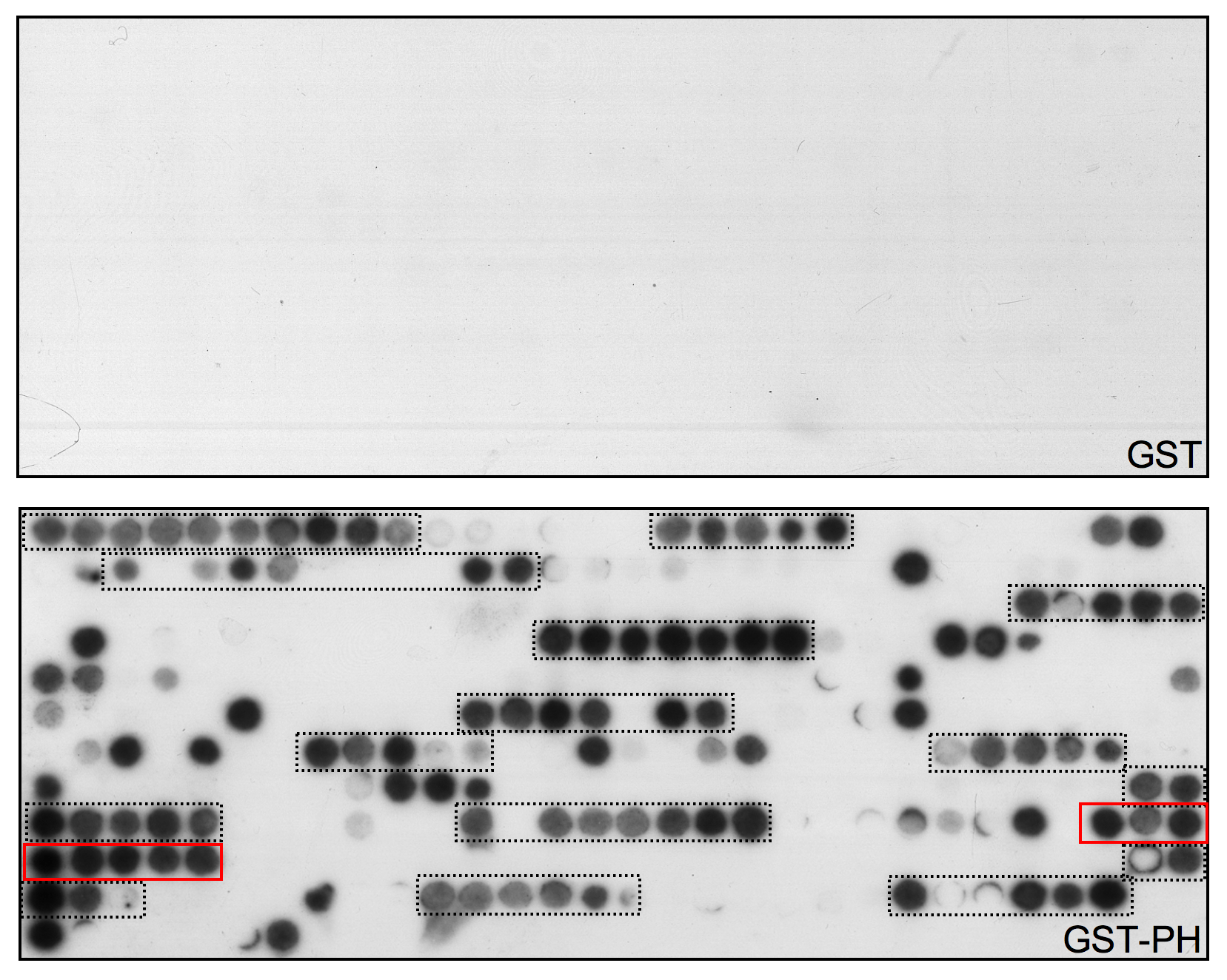

Supplement: Figure S1 — Gab1 peptide array overlay assay identifies potential binding sites for the PH domain. For this assay, the full amino acid sequence of Gab1 from Mus musculus used in the study of Eulenfeld and Schaper [25] was chemically synthesized as an array of spots of overlapping peptides (Multipep synthesiser [Intavis], with a peptide length of 23 amino acids, sliding two residues further with each consecutive peptide), blocked with 5% nonfat dry milk in TrisHCl buffer (pH 7.5) with 100 mM NaCl and 0.1% Tween 20 added and probed initially with 4 µg/ml GST, followed by incubation with anti-GST, HRP-coupled secondary antibody, and ECL detection. No GST binding was detectable to any of the peptides (top panel). The same membrane was then re-probed with 1 µg/ml of affinity-purified GST-PH domain (bottom panel). Series of dark spots correspond to clusters of nonidentical, overlapping peptides that bind to the GST-PH probe. The red box indicates the Ser552 epitope previously implicated in regulating Gab1 PH domain binding by the work of Eulenfeld and Schaper [25]. Similar results were also obtained when DTT was included in the assay to eliminate potential artefacts from non-specific interactions of Cys residues (unpublished data). (1.68 MB TIF) [file pbio.1000591.s001.tif]

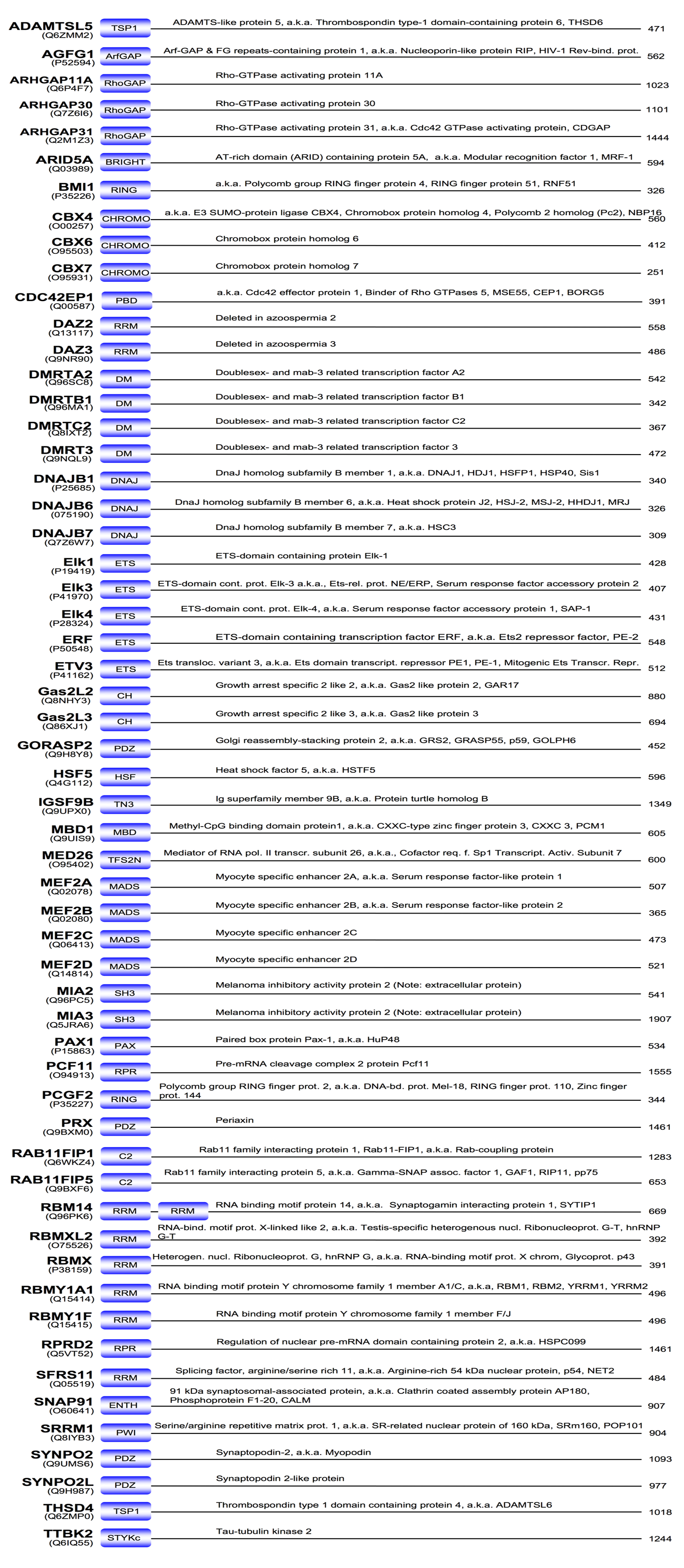

Supplement: Figure S2 — Human proteins identified as NFN candidates by bioinformatics analysis. Schematic representation of proteins identified by the prediction of disordered regions and structural domains for all human proteins in the UniProt SwissProt database (http://www.ebi.ac.uk/uniprot/) using DisEMBL (http://dis.embl.de/) and SMART (http://smart.embl-heidelberg.de/), respectively. The two sets of predictions were compared using a custom perl script to identify proteins with a predicted domain or domains in the N-terminus (defined as the first 25% of the protein), no predicted domains in the C-terminus (defined here as the remaining 75% of the protein), and predominantly disordered (>80%) in this C-terminus. Initial hits were listed with their corresponding SMART and SwissProt data and then individually inspected to exclude, for example, transmembrane proteins. Proteins shown here clearly represent an underestimate of actual candidates in the human proteome, since, for example, proteins with additional domains in the amino acid chain following the folded N-terminus were excluded, even if several hundred disordered amino acids follow the N-terminal domain. If multiple splice variants occur, only a single representative is shown for each protein. Proteins are alphabetically listed according to the gene names following the HGNC nomenclature (July 2010; http://www.genenames.org/), identifiers below the names and SNTD designations are according to the SMART database. Protein domains and chain lengths are not drawn to scale. Values on the right side indicate the number of amino acids in each protein. Many of the NFN candidates depicted here are known or suspected to act in cell signalling. Please note that LMD proteins already depicted in Figure 1 and again found in the bioinformatic analysis (FRS2, FRS3, GAB1, GAB2, GAB3, IRS1, IRS2) are not shown again in this supporting figure. (1.98 MB TIF) [file pbio.1000591.s002.tif]
